# Review of Stimuli-Responsive Polymers in Drug Delivery and Textile Application

**DOI:** 10.3390/molecules24142547

**Published:** 2019-07-12

**Authors:** Sudipta CHATTERJEE, Patrick Chi-leung HUI

**Affiliations:** Institute of Textiles and Clothing, The Hong Kong Polytechnic University, Hung Hom, Hong Kong

**Keywords:** stimuli-responsive polymer, thermo-responsive, pH-responsive, hydrogel, drug delivery, textile

## Abstract

This review describes some commercially available stimuli-responsive polymers of natural and synthetic origin, and their applications in drug delivery and textiles. The polymers of natural origin such as chitosan, cellulose, albumin, and gelatin are found to show both thermo-responsive and pH-responsive properties and these features of the biopolymers impart sensitivity to act differently under different temperatures and pH conditions. The stimuli-responsive characters of these natural polymers have been discussed in the review, and their respective applications in drug delivery and textile especially for textile-based transdermal therapy have been emphasized. Some practically important thermo-responsive polymers such as pluronic F127 (PF127) and poly(*N*-isopropylacrylamide) (pNIPAAm) of synthetic origin have been discussed in the review and they are of great importance commercially because of their in situ gel formation capacity. Some pH-responsive synthetic polymers have been discussed depending on their surface charge, and their drug delivery and textile applications have been discussed in this review. The selected stimuli-responsive polymers of synthetic origin are commercially available. Above all, the applications of bio-based or synthetic stimuli-responsive polymers in textile-based transdermal therapy are given special regard apart from their general drug delivery applications. A special insight has been given for stimuli-responsive hydrogel drug delivery systems for textile-based transdermal therapy, which is critical for the treatment of skin disease atopic dermatitis.

## 1. Introduction

Hydrogels are three-dimensional polymeric networks made up of the same or different hydrophilic polymers and contain large amounts of water in their structure [1,2,3]. The polymeric backbone with hydrophilic functional groups hold a large amount of water in the structure and crosslinking network formed by polymeric chains, which resists its dissolution in water [4,5]. Hydrogels exhibit the ability to swell and possess some degree of flexibilities, which are very similar to the natural tissue due to their large water content. The compactness of hydrogels in aqueous media is maintained by physical cross-linking (e.g., entanglements, crystallites) and, also, chemical cross-linking [6,7]. Hydrogels widely vary in their composition, chemical structure, biodegradability, and various physio-chemical properties such as spectral and mechanical properties, pH stability, and, depending on all such factors, their biological function and performances vary in multiple dimensions [8,9,10]. Hydrogels resemble some physical properties of living tissues such as high-water content, compactness, and low interfacial tension with aqueous media, and those properties of hydrogels impart their great applicability in the bio-medical field, especially for drug delivery [11,12,13,14]. The biomedical applications of hydrogels including drug delivery, tissue engineering, clinical bandages, and biosensors come in the form of film/film forming systems, micro/nano-sphere, matrix, nanoparticles, and nanocomposites depending on the chemical composition and processing parameters of hydrogels [15,16,17,18]. The suitable use of hydrogels for delivering drugs can be upgraded by modifying their transport properties for drugs and also by chemical modification [4,19].

**Figure 1:**
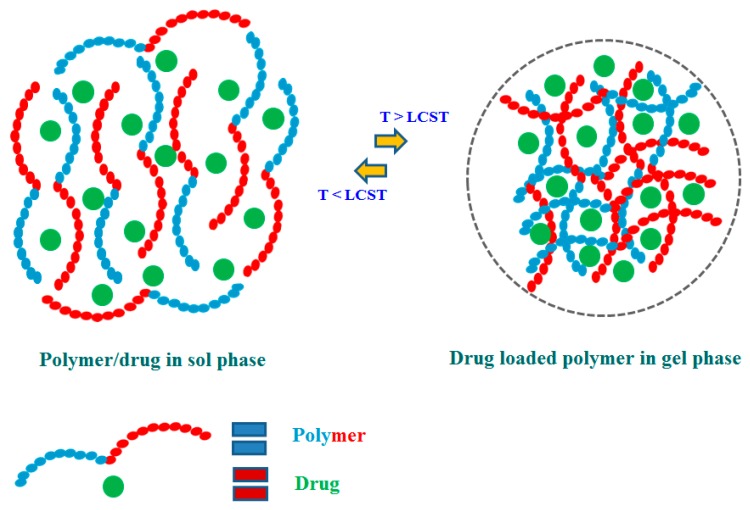
The schematic representation of thermo-responsive behavior (LCST) of the stimuli-responsive polymer hydrogel drug delivery system.

**Figure 2:**
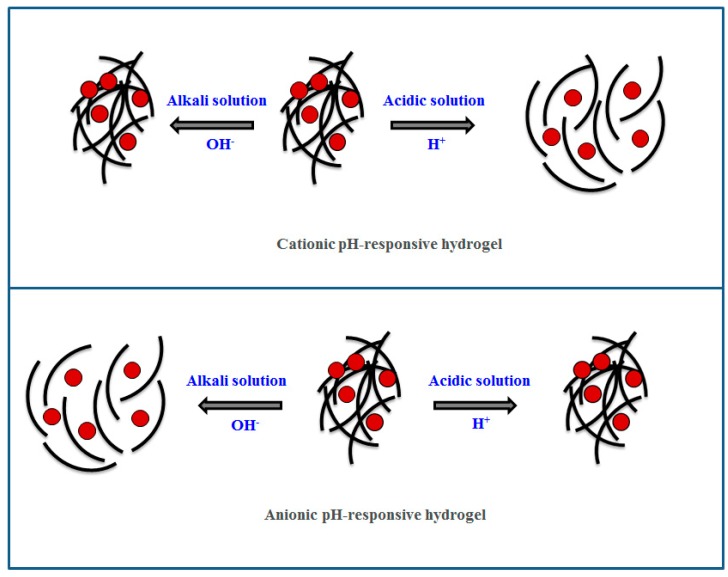
The schematic representation of pH-responsive behavior of the stimuli-responsive polymer hydrogel drug delivery system.

The recent focus on functional polymers mostly centers around stimuli-responsive polymers and these polymers have attracted great attention since these can show sol-gel transitions in response to external triggers like temperature, pH, and light [20,21]. Moreover, the physical changes in stimuli-responsive polymers are reversible and they are capable of returning to their initial state after the trigger is removed [21]. In recent years, there has been a remarkable growth in the development of stimuli-responsive polymer-based drug delivery systems with sustained and controlled drug release properties [22,23]. The important feature of stimuli-responsive polymers is the critical solution temperature (CST), which can be defined as a critical temperature at which the polymeric solution shows a phase separation, which moves from the isotropic state to the anisotropic state [21]. The polymer with lower critical solution temperature (LCST) shows the solution phase below CST and becomes insoluble or forms hydrogels over CST with heating [20]. The LSCT can be defined as a critical temperature at which the polymeric solution shows a phase separation, which moves from the isotropic state to the anisotropic state [21]. The polymers with LCST are mostly used for developing drug delivery systems [22]. Stimuli-responsive gelling materials constructed from natural and synthetic polymers are successfully used to develop drug delivery systems and regenerative medicine products because of their triggered actions in response to external stimuli [24,25,26]. The hydrogels made of stimuli-responsive polymers can show some physical changes like porosity, swelling with external stimuli, and the switchable physical properties provide huge and wide-spread biomedical applications of these polymers [27,28]. Thermo-responsive polymers show sol-gel transitions with a change in temperature of the external environment and hydrogels made of thermoresponsive polymers find tremendous drug delivery applications through simple chemical modifications [29,30,31,32], and some thermoresponsive polymers are capable of forming hydrogel near body temperature [33,34]. A part of thermoresponsive polymers show Arrhenius-type viscosity changes based on coil to globule transitions and others show enthalpic or entropic driven sol-gel transition [22,24]. The synthetic thermoresponsive polymers mainly poly(*N*-isopropylacrylamide) (pNIPAAm) and pluronic F127 (PF127) are widely used for drug delivery applications since they can form gels near the body temperature of 37 °C [35,36,37]. The stimuli-responsive behavior of thermoresponsive polymers has been schematized in Figure 1 and this diagram of the thermoresponsive polymer having LCST shows sol-gel transition with the temperature. The phase change of thermoresponsive polymers in Figure 2 has been shown to be reversible where the polymer chains form hydrogel above LCST and the polymer chains are found to be in close proximity encapsulating a drug within it. The hydrogel reverts back to the sol phase when the temperature is below LCST and the polymers are soluble in the solvent mixture with no encapsulated drug (Figure 2). The swelling-shrinking behavior of pH-responsive polymers in response to an external pH condition makes them suitable for drug delivery applications [38,39,40]. The cationic pH responsive polymers including chitosan, poly(*N*,*N*-dimethylaminoethyl methacrylate) (PDMAEMA), and poly(*N*,*N*-diethylaminoethyl methacrylate) (PDEAEMA) swell in acidic pH and shrink in basic pH, where the basic pH-responsive polymers such as albumin and polyacrylic acid (PAA) swell in basic pH and shrink in acidic pH [41,42]. The stimuli-responsive behavior of pH-responsive polymers has been presented in Figure 2 and this schematic diagram of pH-responsive hydrogels gives an idea about the sensitivity of pH-responsive polymers toward different external pH conditions. As shown in Figure 2, depending on the charge groups of pH-responsive polymers, their hydrogels are found to change their swollen/shrinkage state in response to external pH of the media.

**Figure 3:**
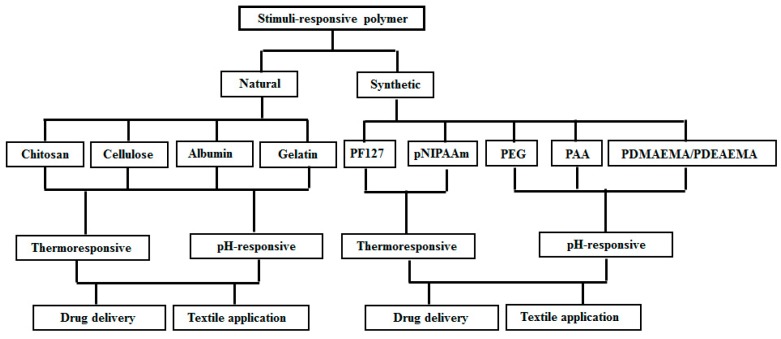
The flowchart outlining the selection of stimuli-responsive polymers and their applications.

**Figure 4:**
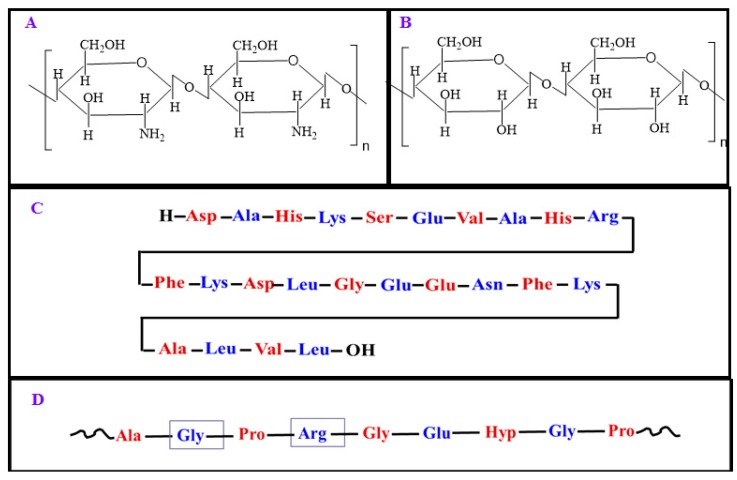
The chemical structures of the natural polymers, chitosan (**A**), cellulose (**B**), serum albumin (**C**), and gelatin (**D**).

**Figure 5:**
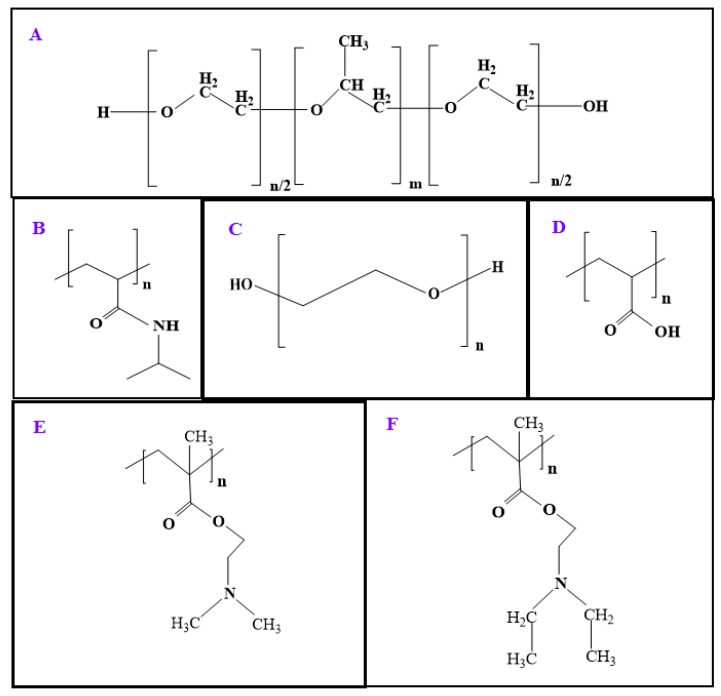
The chemical structure of the thermo-responsive synthetic polymers, PF127 (**A**), pNIPAAm (**B**), and the pH-responsive synthetic polymers, PEG (**C**), PAA (**D**), PDMAEMA (**E**), and PDEAEMA (**F**).

Textile-based transdermal therapy using drug loaded hydrogels made of stimuli-responsive polymers has become an effective means of skin care [35,43,44,45]. Wang et al. [43,44] developed PF127-based thermo-responsive hydrogels to load water soluble traditional Chinese drug Cortex moutan, which has been reported to be effective against pathogenesis related to atopic dermatitis and the transdermal therapy using their system seems to be an effective means to fight against this disease using smart textiles. The functionalized textiles coated with stimuli-responsive hydrogel can balance moisture on the skin and give comfort by actively balancing body temperature [46,47]. The functionalized textiles coated with stimuli-responsive hydrogels are enriched with soft display, aesthetic appeal, and improved wetting properties [48,49,50]. In this review, the drug delivery and textile applications of various stimuli-responsive polymers have been considered. The textile applications of stimuli-responsible polymers mainly focus on textile based transdermal therapy where drugs and moisture are simultaneously transferred to infected sites on the skin. The whole review has been divided into two parts: drug delivery and textile applications of natural stimuli-responsive polymers including chitosan, albumin, cellulose, gelatin, and those of synthetic polymers including PF127, pNIPAAm, poly(ethylene glycol) (PEG), polyacrylic acid (PAA), and poly(dimethylaminoethyl methacrylate) (PDMAEMA) and poly(diethylaminoethyl methacrylate) (PDEAEMA). Figure 3 highlights the main objective of the study and outlines the properties of stimuli-responsive polymers of biological and synthetic origin, and their biomedical applications in brief. The chemical structure of natural polymers are given in Figure 4. Figure 5 depicts the chemical structure of all synthetic polymers described in this case. The natural polymers are capable of showing both thermo-responsive and pH-responsive properties as well as simple chemical modifications and composite formation with other polymers are applied to increase and improve their biomedical applications especially for drug delivery. Synthetic thermo-responsive polymers are well recognized for their in situ gel forming ability. Their simple chemical modifications and composite formations are applied using other polymers of natural or synthetic origin to increase their biocompatibility and reduce toxicity. The chemical nature, type of stimuli-responsiveness, and biomedical applications of all stimuli-responsive polymers are described in Table 1.

## 2. Natural Stimuli-Responsive Polymers

### 2.1. Chitosan

Chitosan as a natural stimuli-responsive polymer is capable of showing both thermo-responsive and pH responsive properties [51,52]. The drug delivery systems made of chitosan show relevance to drug delivery applications especially for cancer therapy and skin treatment due to their biocompatibility, biodegradability, and low toxicity [24]. The drug delivery system made of chitosan, hyaluronic acid, and pNIPAAm showed thermo-responsive property and was used for controlled delivery of analgesic drug nalbuphine [53]. The thermo-responsive hydrogel made of cross-linked chitosan and pNIPAAm via emulsion polymerization was applied as a drug delivery system for antibacterial drug levofloxacin [54]. The thermo-responsive hydrogel made by grafting pluronic onto chitosan was used as an injectable cell delivery system for cartilage regeneration [55]. The injectable thermo-reversible hydrogel made from PEG grafted chitosan was used for sustained release of drugs and the hydrogel system also showed a pH-responsive property [56]. The drug delivery system made from chitosan and αβ-glycerophosphate exhibited a thermo-responsive property and was used as a drug delivery system for adriamycin and 6-mercaptopurine [57]. The thermo-responsive hydrogel made of pNIPAAm and chitosan was applied on cotton fabric using glutalraldehyde as a cross-linker to impart antibacterial activity to cotton fabric [58].

Chitosan due to its primary amine groups forms a cationic hydrogel network in water and it shows pH-responsive behavior by swelling in acidic pH (pH < pKa) and shrinking in basic pH (pH > pKa) [59]. The hydrogel formed from chitosan, acrylic acid, (2-dimethylamino) ethyl methacrylate via in situ free radical polymerization showed pH-responsiveness with enhanced mechanical stability and was used as a drug delivery system for controlled delivery of bovine serum albumin and 5-fluorouracil in cancer therapy [60]. The bone ash-reinforced chitosan-based pH-responsive hydrogel loaded with model drug amoxicillin was applied for the treatment of gastric ulcer [61]. The hydrogel structure was fabricated by photopolymerization of chitosan-grafted-glycidyl methacrylate and poly(ethylene glycol)diacrylate under UV light [61]. The hydrogel with pH responsive character was developed from chitosan and poly(ethylene oxide) and applied for oral delivery of metronidazole and amoxicillin [62]. The pH-responsive hydrogel network was found to release more drugs in simulated gastric fluid than simulated intestinal fluid [62]. The pH-responsive hydrogel made of chitosan and poly(*N*-vinyl-2-pyrrolidone) in the presence of 74% neutralized PAA was used for wound healing. It showed different extent of swelling depending on the pH of external media [63]. The pH-responsive character of chitosan hydrogels was imparted on textile fabric by coating or integrating on it to develop smart textiles or medical textiles [50,64].

### 2.2. Cellulose

Cellulose, which is the most abundant natural polymer on earth, is made up of glucose monomers and capable of forming hydrogel using their functional hydroxyl groups [65,66]. Methylcellulose, which is the functional material developed from cellulose, is water soluble and can show a thermo-responsive property with the sol-gel transition in the temperature range of 60 to 80 °C [24,67]. The thermo-responsive hydrogel made from pNIPAAm and methylcellulose showed gel formation near the body temperature and the mechanical strength of the hydrogel was improved [68]. The thermo-responsive hydrogel from methylcellulose and PF127 was used as a drug delivery system for anti-cancer drug docetaxel and the system showed more sustained drug release than free docetaxel [69]. The thermo-responsive hydrogel from kappa-carrageenan and methylcellulose showed double thermal gel-sol-gel transition upon heating and was used for developing the drug delivery system [70]. The injectable bone substitute was prepared from the thermo-responsive hydrogel of methylcellulose, gelatin, and citric acid with a bio-ceramic powder loaded into it [71]. The injectable thermosensitive hydrogel from methylcellulose and chitosan was applied for tissue engineering and showed sol-gel transition near the body temperature of 37 °C [72]. The carbomethyl cellulose, which is another functional derivative of cellulose, is capable of forming thermo-responsive hydrogel. The drug delivery system formed from carboxymethyl cellulose and gelatin showed sol-gel transition near the body temperature and was used for transdermal drug therapy with lidocaine [73]. The thermo-responsive hydrogel formed from carboxymethyl cellulose sodium and Poloxamer 407 was used as the drug delivery system for Chinese herbal medicine in textile-based transdermal therapy [43,44]. The hydrogel system with traditional Chinese medicine was mainly applied for the treatment of atopic dermatitis and the hydrogel with thermo-responsive property is capable of showing sol-gel transition near a body temperature of 37 °C [43,44].

The stimuli-responsive hydrogels made from cellulose have been becoming popular for the last few years due to their biocompatibility and non-toxicity. The pH-responsive hydrogel of cellulose was developed by simply mixing aqueous solutions of cellulose acetoacetate and cystamine dihydrochloride at room temperature and, moreover, this cellulose-based hydrogel showed dual responsiveness via reversible sol-gel transitions in response to both pH and redox triggers [74]. The cellulose-based hydrogels after epichlorohydrin mediated cross-linking of quaternized cellulose and carboxymethyl cellulose showed a pH responsive property along with salt responsiveness and the mass ratio of two cellulose-based compounds in the composites was varied to compare their pH and salt responsiveness [75]. These cellulose-based hydrogels are reported to have various biomedical applications including drug delivery and tissue engineering [75]. The hydrogel system made of carboxymethyl cellulose and pNIPAAm showed the pH-responsive character along with thermo and redox responsiveness [76]. The multi-responsive hydrogel was used as a drug delivery system for lysozyme and the release of lysozyme from the system was studied at a different pH, temperature, and redox environments to ensure its maximum effective drug delivery applications [76]. The pH-responsive hydrogel made of hydroxyethyl cellulose and hyaluronic acid was used as a drug delivery system for the treatment of skin lesions and this transdermal drug delivery system loaded with isoliquiritigenin was found to be effective for the treatment of acne since it showed excellent permeability in the skin [77].

### 2.3. Albumin

Albumin, which is a natural globular protein commonly found in blood plasma, is capable of showing a stimuli-responsive property in response to external stimuli like temperature and pH [24]. The thermo-responsive hydrogel made from bovine serum albumin (BSA) and low molecular weight polyethylene glycol (PEG) showed sol–gel transition near the body temperature and it was reported to be a promising drug delivery material [78]. The pH responsive hydrogel developed by free radical polymerization of methacrylate bovine serum albumin and *N*-isopropylacrylamide was used as a drug delivery system for caffeine and theophylline [79]. The curcumin-loaded thermo-responsive nano-hydrogel was developed with BSA for ophthalmic treatment [80].

The anionic hydrogel forming albumin can show pH-responsiveness by swelling in basic pH medium and shrinking in acidic pH [27,81]. The pH-responsive hydrogel developed from BSA with hydroxyethyl methacrylate and acrylic acid monomers was used as a drug delivery system for anti-cancer drug flutamide [82]. BSA-based pH responsive hydrogel formed by free radical polymerization was applied as a drug delivery system for oral delivery [83]. The drug delivery system based on the pH-responsive hydrogel from alginate and albumin was used for delivery of prednisolone [84]. BSA-based pH-responsive hydrogels were coated on textile fabric to develop medical textiles for wound healing [85].

### 2.4. Gelatin

Gelatin, which is an animal origin natural protein, can show thermo-responsive properties and is commercially available in different form such as hydrogels, fibers, and nanoparticles [86]. The tunable thermo-responsive gelatin hydrogel showed volume transition near the body temperature and it was reported to be a promising drug delivery system [87]. The injectable thermo-responsive hydrogel made of chitosan and gelatin showed sol-gel transition near the body temperature and it showed compatibility with human tissue [88]. The thermo-responsive hydrogel system developed from chitosan, gelatin, and glycerol phosphate was applied as a cell carrier for nucleus pulposus regeneration [89]. The thermo-responsive hydrogel of gelatin and chondroitin sulphate was obtained by a simple method involving layer-by-layer (LbL) deposition and the system was developed with high mechanical strength, which improved its biomedical applications [90]. The thermo-responsive hydrogel made of carboxymethyl cellulose and gelatin copolymer was used as a drug delivery system with lidocaine in transdermal drug delivery and the system was used for percutaneous delivery of the drug [73].

The nanogel made of gelatin, *N*,*N*′-ethylenebisacrylamide, and sodium methacrylate showed a pH-responsive character and it was developed by a solvent-free emulsion polymerization method with sun flower seed oil as a continuous phase [91]. The gelatin-based nanogel was used as a drug carrier for diclofenac sodium salt [91]. The pH-responsive hydrogel was developed from gelatin and acrylic acid for colon-specific oral drug delivery using ketoprofen as a model drug [92]. The pH-responsive hydrogel of gelatin methacrylate was developed for the controlled delivery of gentamicin and ampicillin (Amp) and both drugs loaded into the hydrogel showed synergistic activity in killing multi drug resistant bacteria [93]. The gelatin-based pH-responsive hydrogel was developed by grafting β-cyclodextrin to gelatin (Gel), which is followed by cross-linking with oxidized dextran and the hydrogel was used as a drug delivery system for anti-cancer drug 5-fluorouracil [94].

## 3. Synthetic Stimuli-Responsive Polymers

### 3.1. Pluronic F127

Pluronic F127 (PF127) is a non-ionic triblock copolymer of poly(ethylene oxide)-*b*-poly(propylene oxide)-*b*-poly(ethylene oxide) (PEO-PPO-PEO) and, being a thremo-responsive synthetic polymer, PF127 can form the hydrogel near the body temperature [35,95]. PF127 is approved by the ‘U.S. Food and Drug Administration’ (FDA) to be used in biomedical areas especially for drug delivery, tissue engineering, and the administration of the PF127-based drug delivery system, which can be oral, ocular, intranasal, subcutaneous, vaginal, and rectal [95]. For last few years, the potential drug delivery applications of PF127-based systems have been emerging for transdermal therapy [35]. The thermo-responsive hydrogel made of PF127 and hyaluronic acid showed gel formation at a body temperature of 37 °C and was used as a drug delivery system for a human growth hormone [96]. The thermo-responsive hydrogel made of hyaluronic acid grafted PF127 showed in situ gel formation and was applied for delivery of model anti-cancer drugs cisplatin and carboplatin [97]. The drug delivery system made from thermo-responsive hydrogel of glycol chitosan and PF127 by photo-polymerization was applied for delivery of doxorubicin [98]. The biodegradable thermo-responsive composite hydrogel made of PF127 and PEG-poly(ε-caprolactone)-PEG copolymer was applied as a drug delivery system for model drugs Vitamin B_12_, honokiol, and BSA and the hydrogel showed sol-gel transition at the body temperature [99]. The thermo-responsive hydrogel made from chitosan and PF-127 was applied as a drug delivery system for model anti-cancer drug 5-fluorouracil [100]. The hydrogel system made of PF127 and Pluronic F68 was evaluated for in situ hydrogel formation under physiological conditions and the hydrogel made of PF127 (20 wt%) was found to be capable of forming hydrogel under a physiological condition. However, the addition of Pluronic F68 to PF127 enhanced the gelation temperature and could not form hydrogels under physiological condition [101]. This thermo-responsive hydrogel was applied for ocular drug delivery [101]. The thermo-responsive hydrogel made of PF127 and alginate was applied as a drug delivery system of a model drug selegeline for transdermal therapy. The drug delivery system showed sustained and controlled release of selegeline [102]. The PF127-based thermo-responsive hydrogels were used in textile-based transdermal therapy for the treatment of AD. The hydrogels made of PF127 and carboxymethyl cellulose sodium were loaded with Chinese herbal medicine to deliver moisture and drugs simultaneously to infected sites on the skin [43,44].

### 3.2. Poly(N-isopropylacrylamide)

Poly(*N*-isopoprylacrilamide) (pNIPAAm), which is a synthetic thermo-responsive polymer can form hydrogel at or near the body temperature [103]. pNIPAAm is the mostly studied and FDA-approved, widely applied thermo-responsive polymer for drug delivery applications [24,104,105]. The thermo-responsive hydrogel made of pNIPAAm and PAA showed sol-gel transition near the body temperature and was used as a drug delivery system [106]. The hydrogel system also showed a pH-responsive property [107]. The thermo-responsive hydrogel made of pNIPAAm and methylcellulose showed gel formation at the body temperature and was used as a promising drug delivery system near the body temperature [68]. The hydrogel from pNIPAAm and butyl methacrylate (BuMA) showed a thermo-responsive property with zero order drug release profiles [108]. The thermo-responsive hydrogel made of pNIPAAm and alginate showed gel formation near the body temperature and was used as a drug delivery system for anti-cancer drug doxorubicin [109]. The thermo-responsive hydrogel made of pNIPAAm and cellulose nanocrystals via free-radical polymerization showed gel formation within a range of 36 to 39 °C and was loaded with model drug metronidazole for a wound dressing purpose [110]. The thermo-responsive hydrogel made of pNIPAAm and PEG-diacrylate showed in situ gel formation and was applied for ocular drug delivery with model drugs immunoglobulin-G and BSA [111]. The thermo-responsive hydrogel of pNIPAAm and spiropyran formed by a facile and versatile surface-initiated controlled polymerization method (SI-ARGET-ATRP) showed the capability of dimensional changes on cotton fabric upon irradiation with visible light or a temperature stimulus [112]. The thermo-responsive nano-hydrogel made of pNIPAAm and chitosan was applied on cotton fabric as a surface modifying system and the nanohydrogel also showed pH-responsiveness [113]. Thermo-responsive pNIPAAm was grafted onto cotton fabric to modify the surface property of the fabric, which was dependent on the temperature during the grafting process, and this was applied to develop smart textiles for clinical applications [114].

### 3.3. Poly(ethylene glycol) (PEG)

Poly(ethylene glycol) (PEG) is a water-soluble synthetic polymer with excellent biocompatibility and no-toxicity, and shows a pH-responsive property in response to an external pH change [115]. The pH-responsive hydrogel made of the co-polymeric network of PEG and poly(methacrylic acid) was used for oral delivery of model drug oxaliplatin and it was used for colon targeting of the drug [116]. The pH-responsive hydrogel made of the PEG derivative and α,β-polyaspartylhydrazide was applied as a drug delivery system for anti-cancer drug doxorubicin [117]. The drug delivery system made of PEG and cholesterol-modified poly(monomethyl itaconate) showed a pH-responsive property and was used for controlled and targeted release of the model drug piroxicam, which was used in tumor-targeting chemotherapy [118]. The pH-responsive hydrogel from PEG and poly(itaconic acid) was developed using UV-initiated free radical polymerization with tetraethylene glycol as the cross-linking agent and Irgacure 2959 as the initiator and it was used as a drug delivery system for oral delivery of the drug [119]. The pH-responsive nanoparticles made of PEG-poly(l-histidine)-poly(l-lactide) were used as drug carriers for anti-tumor drug doxorubicin [120]. The pH-responsive micellar drug delivery system made of acetal-linked poly(ethylene glycol)-block-polylactide copolymer was applied for delivery of anti-cancer drug paclitaxel [121]. The pH-responsive hydrogel made of PEG and chitosan was applied on the cotton membrane to develop medical textiles for wound dressing [122].

### 3.4. Polyacrylic Acid

Polyacrylic acid (PAA), which is an anionic synthetic compound, can form pH-responsive hydrogel, swells/dissolves at pH higher than its pKa, and remains collapsed at acidic pH (pH < pKa) [123]. This pH responsive behavior of PAA attributes to oral delivery of the drug by the PAA-based drug delivery system [124]. The pH-responsive anionic hydrogels made of PAA can protect drugs from degradation and denaturation at a low pH of gastric juice and release drugs in specific locations, such as the upper small intestine and colon [125]. The pH-responsive PAA-based hydrogel cross-linked by poly(l-glutamic acid)-g-(2-hydroxyl methacrylamide) showed pH-dependent swelling-shrinking behaviors and was used for the delivery of the model protein BSA [126]. The pH and thermo-responsive micelles developed from the block copolymer of PAA and pNIPAAM (pNIPAAm-*b*-PAA) was used as a drug delivery system for anti-cancer drug doxorubicin [127]. The pH-triggered oral drug delivery system was developed by capping mesoporous silica SBA-15 with pH-responsive polymer PAA via a facile graft-onto strategy and it was used to deliver anti-cancer drug doxorubicin for the treatment of colon cancer [128]. The pH-responsive biodegradable hydrogels made from four types of pH-sensitive PAA derivatives and poly(l-glutamic acid) as a cross-linker were applied for oral delivery of insulin [129]. The pH-responsive hydrogel made of polyvinyl acetate cross-linked PAA was applied on textile fabric to develop wound healing monitoring textiles [130].

### 3.5. Poly(N,N-dialkylaminoethyl methacrylate) and Eudragit

Poly(*N*,*N*-dimethylaminoethyl methacrylate) (PDMAEMA) and poly(*N*,*N*-diethylaminoethyl methacrylate) (PDEAEMA), which are both cationic pH-responsive polymers and swell in acidic pH (pH < pKa) due to the protonation of their tertiary amine groups [131]. The pH-responsive hydrogels formed by PDMAEMA and PDEAEMA find drug delivery applications since they can change the swelling-deswelling state in response to change in body pH, especially in the gastro-intestinal environment of the human body [132]. The pH-responsive hydrogel made of block co-polymer of poly(dimethysiloxane) and PDMAEMA was applied to deliver anticancer drug doxorubicin and the block co-polymer (poly(dimethysiloxane)-*b*-PDMAEMA) was synthesized by atom transfer radical polymerization (ATRP) [133]. The pH-responsive hydrogel made of poly(vinyl alcohol) and PDMAEMA was used for drug delivery applications [134]. The structural hydrogels having a semi-interpenetrating network with different molecular weights were used to deliver riboflavin and PDMAEMA played a vital role in the swelling under different pH conditions [134]. The pH-responsive nano-hydrogel was synthesized by copolymerization of PDEAEMA with hetero-bifunctional PEG bearing a 4-vinylbenzyl group at one end and a carboxylic acid group at the other end. The nano-hydrogel was used as a drug delivery system for anti-cancer drug doxorubicin [135]. The pH-responsive micelles from the copolymers of ethyl cellulose-graft-PDEAEMA through atom transfer radical polymerization were applied as the drug delivery system for model drug rifampicin [136]. The pH-responsive PDMAEMA was grafted onto the cotton surface to develop medical textiles for low adherent wound dressing [137].

**Figure 6:**
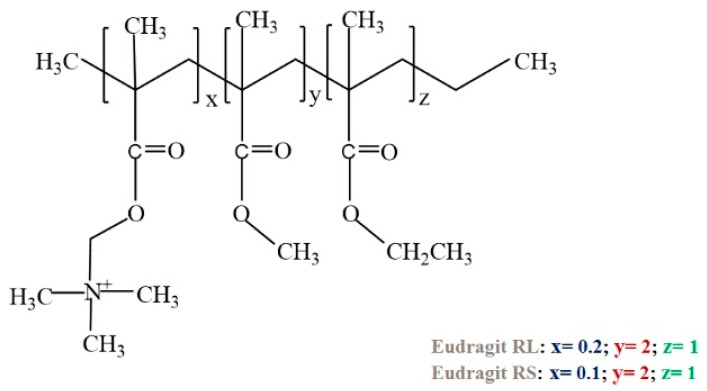
The chemical structure of Eudragit RL and RS.

Eudragit prepared by the polymerization of acrylic and methacrylic acids or their esters was first introduced commercially in 1950 by Röhm GmbH & Co. KG—Germany. Eudragit RL and Eudragit RS are anionic copolymers of acrylic and methacrylic acid esters having quaternary ammonium groups and used as anionic pH-responsive polymers for mainly oral delivery of the drug [138]. The chemical structure of Eudragit RL and Eudragit RS has been given in Figure 6. pH-responsive nanoparticles made of Eudragit RL 100 and Eudragit RL 100-poly(lactic-co-glycolic acid) were used as drug delivery systems for diclofenac sodium in an intestinal pH condition of 6.8 [139]. The microspheres made of pH-responsive Eudragit RS100 were used as a drug delivery system for acidic drug ibuprofen [140]. Eudragit S100 as a pH-responsive polymer was coated onto the liposomes by a fast and organic solvent-free method for the colonic delivery of curcumin [141]. The transparent film formed from Eudragit E100 polymer was used for transdermal therapy since it showed good adhesion to the skin [142]. The loaded drug nicorandil was released due to erosion of hydrophilic Eudragit E100 polymer and the release of the drug was observed to be 100% within 20 min [142].

## 4. Conclusions

Some stimuli-responsive polymers have made a significant contribution in the area of drug delivery. The stimuli-responsive polymers of natural origin such as chitosan, cellulose, albumin, and gelation are capable of showing both thermo-responsive and pH-responsive characters. Their stimuli-responsive properties are highly recommended for drug delivery applications, and also in textile-based transdermal therapies. Some synthetic thermo-responsive polymers such as PF127 and pNIPAAm are capable of in situ gel formation, and their drug delivery applications have earned extremely high commercial importance. PF127 is now being successfully applied for transdermal therapy based on textiles. pH-responsive polymers vary in their surface charges, and, depending on the surface charge, their mode and site of applications vary. The pH-responsive polymers are useful for developing drug delivery systems and being used for transdermal therapies. In this review, some natural polymers (chitosan, cellulose, albumin, and gelatin) with both thermo-responsive and pH-responsive characters are discussed and their drug delivery and textile applications are mentioned. The drug delivery and textile applications of some synthetic thermo-responsive (PF127 and pNIPAAm) and pH-responsive polymers (PEG, PAA, and PDMAEMA/PDEAEMA) have been discussed, which gives a special emphasis on their applications in textile-based transdermal therapy. The stimuli-responsive hydrogel-based drug delivery systems are already proven effective for the treatment of skin disease atopic dermatitis via textile-based transdermal therapy.

The stimuli-responsive polymers highlighted in this review are commercially available and their biomedical applications, especially for drug delivery, are well documented. However, their applications in textile-based transdermal therapy need further and more extensive research since many factors like skin pH and temperature come into play, as these factors are found to be varied depending on the physiological state of a person. Furthermore, the stability of drug delivery systems upon contact with skin is a major hurdle since the hydrogels of some thermo-responsive polymers like PF127 completely disintegrate under an acidic condition. Additionally, skin pH around 5.5 is good enough to destabilize the PF127-based hydrogel system. Therefore, more experiments are required to enhance pH stability of such drug carriers for successful application in transdermal therapy via textiles.

## Figures and Tables

**Table 1:** Chemical nature, type of stimuli-responsiveness, and biomedical applications of stimuli-responsive polymers.

Polymer	Chemical Nature	Type of Stimuli-Responsiveness	Biomedical Applications
Chitosan	Natural (polysaccharide)	Thermo-responsive pH-responsive	Drug delivery, tissue engineering, textile application
Cellulose	Natural (polysaccharide)	Thermo-responsive pH-responsive	Drug delivery, tissue engineering, textile application
Albumin	Natural (polypeptide)	Thermo-responsive pH-responsive	Drug delivery, tissue engineering, textile application
Gelatin	Natural (polypeptide)	Thermo-responsive pH-responsive	Drug delivery, tissue engineering, textile application
PF127	Synthetic	Thermo-responsive, *in-situ* gel formation	Drug delivery, textile based transdermal therapy
pNIPAAm	Synthetic	Thermo-responsive, *in-situ* gel formation	Drug delivery, textile application
PEO	Synthetic	pH-responsive (neutral)	Drug delivery, textile application
PAA	Synthetic	pH-responsive (anionic)	Drug delivery, textile application
PDMAEMA/PDEAEMA	Synthetic	pH-responsive (cationic)	Drug delivery, textile application
